# An unusual complication of sacral nerve root injury following bone marrow harvesting: a case report

**DOI:** 10.1186/s12885-019-5567-7

**Published:** 2019-04-11

**Authors:** Tae-Woo Nam, Hyun-Min Oh, Jae-Eun Lee, Ju-Hyun Kim, Jong-moon Hwang, Eunhee Park, Tae-Du Jung

**Affiliations:** 1 Department of Rehabilitation Medicine, Kyungpook National University Hospital, 130 Dongdeok-ro, Jung-gu, Daegu, 41944 South Korea; 2 Department of Rehabilitation Medicine, Kyungpook National University Medical Center, Daegu, South Korea; 3 Department of Rehabilitation Medicine, School of Medicine, Kyungpook National University, 807 Hoguk-ro, Buk-gu, Daegu, 41404 South Korea

**Keywords:** Hematopoietic stem cell transplantation, Bone marrow harvesting, Sacral nerve root injury, Radiculopathy, Electromyography

## Abstract

**Background:**

Hematopoietic stem cell transplantation (HSCT) remains an important therapeutic option for many hematologic malignancies. Bone marrow harvesting from an appropriate donor must be conducted for hematopoietic stem cell transplantation (HSCT). Many previous studies show complications of the recipient after hematopoietic stem cell transplantation (HSCT). However, complications of the donor after bone marrow harvesting are rare. We here report a unique case of a patient who developed sacral nerve root injury after bone marrow harvesting.

**Case presentation:**

A 26-year-old man was admitted to our medical center complaining of acute onset painful burning and tingling sensation at the left posterior thigh and calf. He was a bone marrow donor for his brother’s bone marrow transplantation. He had underwent a bone marrow harvesting procedure two days before admission as a bone marrow donor, using both posterior superior iliac spine (PSIS) as the puncture site.

Pelvic magnetic resonance image (MRI) showed enhancement around the left S2 nerve root in T1 and T2-weighted images. Nerve conduction studies (NCS) revealed normal conduction velocity and amplitude on both lower extremities. Electromyography (EMG) presented abnormal spontaneous activity and neurogenic motor unit potentials on the S2-innervated intrinsic foot muscle and gastrocnemius, soleus muscle on the left.

The patient was treated with pregabalin for pain control. The patient was followed up after 3, 6, and 12 months. Neuropathic pain improved to Visual Analogue Scale (VAS) 1, and recovery state was confirmed by re-innervation patterns of motor unit potentials in electromyography.

**Conclusion:**

Bone marrow harvesting is a relatively safe procedure. However, variable complications may occur. Accurate anatomical knowledge and carefulness are required to avoid sacral nerve root injury when performing the bone marrow harvesting procedure.

## Background

Hematopoietic stem cell transplantation (HSCT) remains an important therapeutic option for many hematologic diseases. Hematopoietic stem cell transplantation (HSCT) was first attempted in 1959, and hematopoietic stem cell transplantation (HSCT) has since become a widely used method as treatment for hematologic diseases [1].

Bone marrow harvesting should be performed on appropriate donors. In bone marrow harvesting, the donor is typically punctured through the posterior superior iliac spine (PSIS) in the prone position, and the bone marrow is collected from the posterior iliac crest. The trajectory of the needle should either be parallel to the iliac crest or perpendicular to the posterior superior iliac spine (PSIS), depending on the harvest site being used [2]. It is an uncomplicated procedure requiring hospitalization of 1–3 days. However, knowledge of aseptic technique and accurate anatomy is needed.

The donation of hematopoietic stem cells (HSCs) is considered to be a safe procedure. Complications that may be present in the donor include aspiration site pain, anemia, vasovagal reaction, infection, and side effects due to general anesthesia. Fatal adverse effect reports are rare [3].

There were no reported complications of sacral nerve root injuries. In this report, we will describe a sacral nerve root injury in the donor, specifically the S2 nerve root injury, after bone marrow harvesting.

## Case presentation

**Fig. 1:**
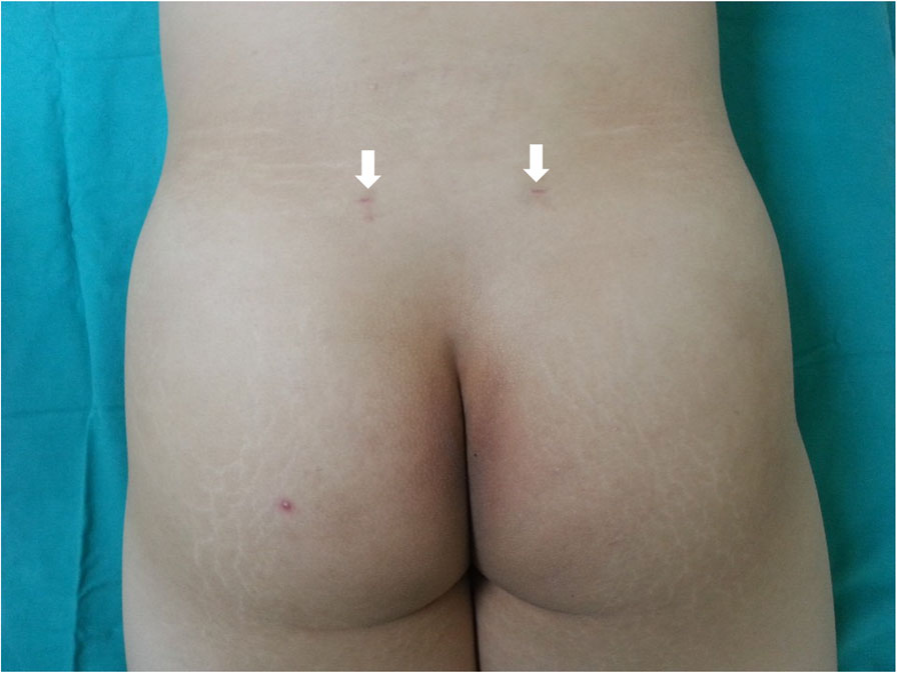
Puncture sites 1 month after conducting bone marrow harvesting (Arrows)

A 26 year-old man was identified to donate marrow for his brother. His height was 178 cm and his weight was 79.2 kg (Body mass index 25.0). He had no bleeding history or other medical problem. Bone marrow harvesting was performed under spinal anesthesia [4]. The patient was put in the prone position, and the bony landmarks of the posterior iliac crest and sacroiliac joint were palpated for the identification of a proper puncture site (Fig. 1). Aspiration trocar and needle were pushed through the skin and subcutaneous tissue to the posterior iliac crest, and the cortical bone was punctured. Bone marrow aspiration was performed after positioning the needle tip within the cortical wall of the posterior crest [2]. There was no repositioing of the needle. The total surgery time was 62 min. A total of 900 cc of bone marrow(450 cc per site) was collected which yielded 1.46 × 10^8^ CD34-positive cells from the two puncture sites shown in Fig. 1. No special problems occurred during the procedure. The donor was hospitalized one more day after bone marrow harvesting to check complications and to control the pain. There was no evidence of hematoma on the puncture sites. While in hospital, he suffered mild pelvic pain which had responded to an oral non-steroidal anti-inflammatory drug (NSAID).

Two days after the bone marrow harvesting, a pain of tingling and stabbing nature appeared on his left posterior thigh and calf. Pain score was noted at Visual Analogue Scale (VAS) 7 points on resting and aggravated with motion. Allodynia was present. Sensory of all dermatome was intact, and no muscle weakness was present. However, there was gait disturbance due to pain.

**Fig. 2:**
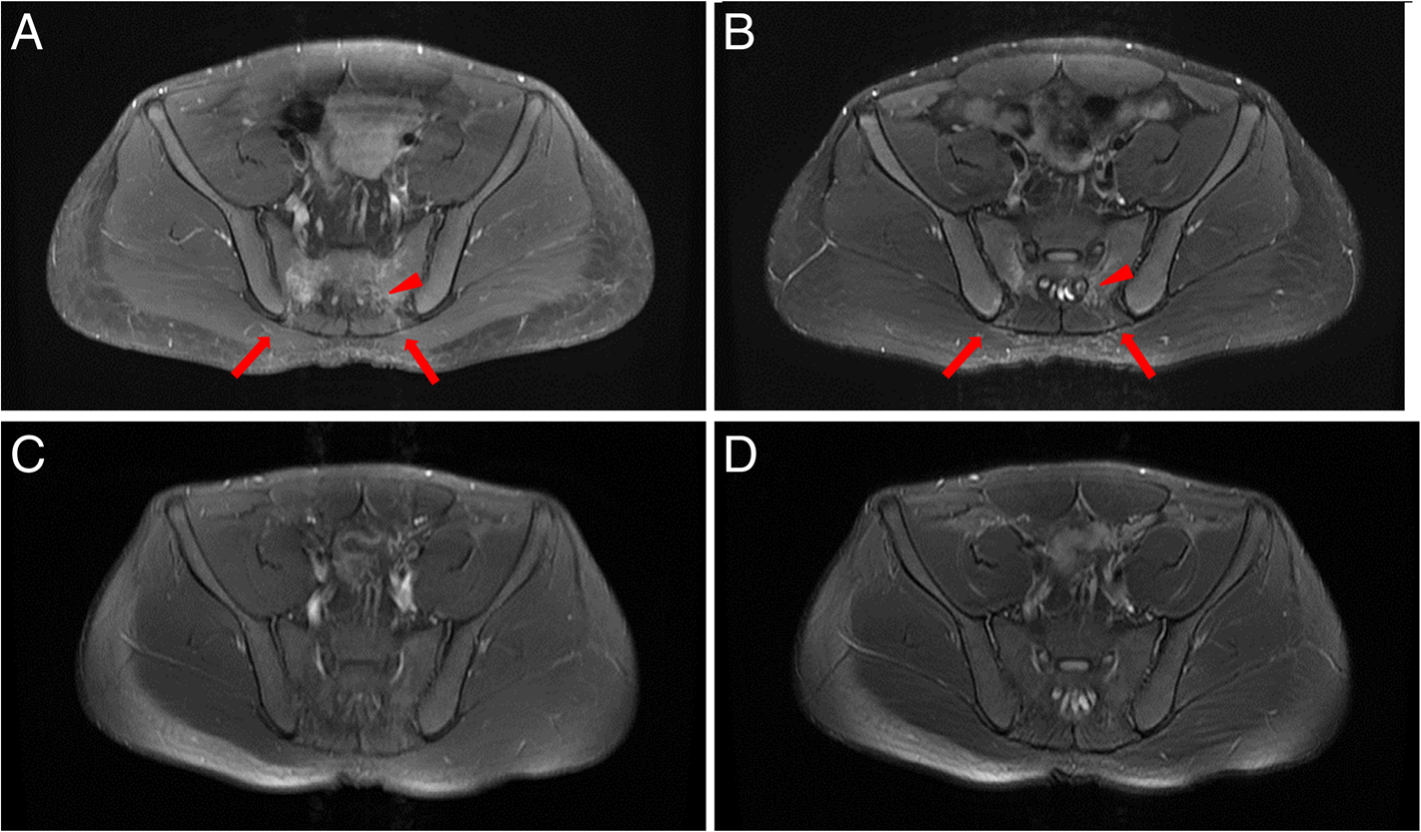
Pelvic magnetic resonance image (MRI) revealed left S2 nerve root injury. Initial T1 weighted image (**a**) and T2 weighted image (**b**) show enhancement around the S2 neural foramen (arrow head) and patchy edematous change with enhancement in the sacrum, retrosacral muscles, and subcutaneous layer around trajectory of the needle (Arrows). T1 weighted image (**c**) and T2 weighted image (**d**) of the follow up pelvic magnetic resonance image (MRI) 3 months after the onset. Little residual enhancement was present around the left S2 nerve root. However, edematous change and enhancement of the muscle and subcutaneous layer were not observed

We conducted a pelvic magnetic resonance image (MRI), nerve conduction study (NCS), and electromyography (EMG) for evaluation. T1 and T2 weighted images of the pelvis magnetic resonance image (MRI) showed patchy edematous change with enhancement in the sacrum, retrosacral muscles, and subcutaneous layer, and the left S2 neural foramen (Fig. 2a, b). Imaging studies indicated that the left S2 nerve root was injured by mechanical damage when the puncture needle was inserted and that the nerve irritation and inflammation were the cause of the patient’s symptoms [5, 6].

**Fig. 3:**
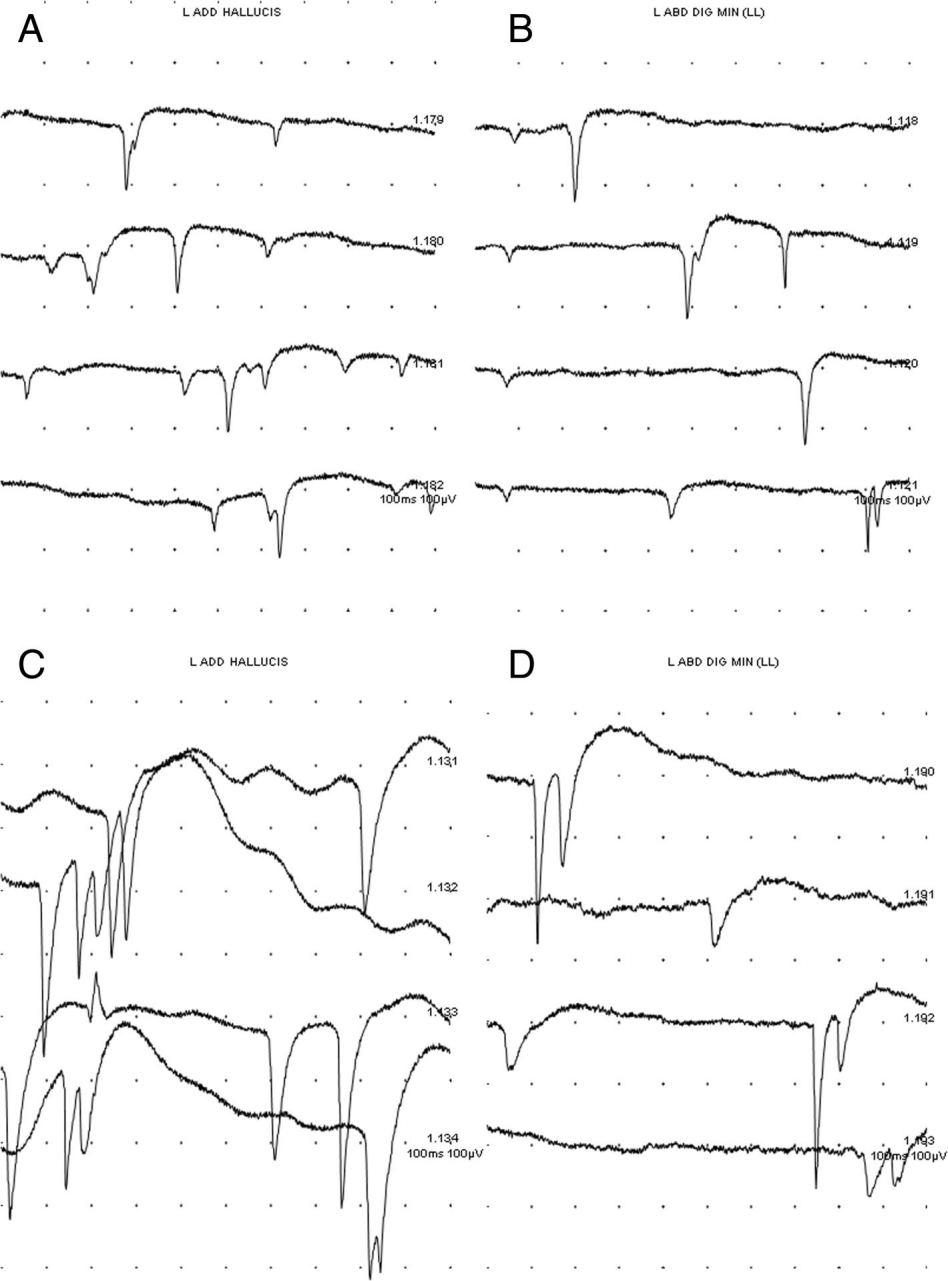
Spontaneous activity on foot intrinsic muscles at 1 month (**a**, **b**) and 3 month (**c**, **d**) since symptom onset. Abnormal spontaneous activities known as positive sharp waves were observed

After 1 month since the pain developed, nerve conduction study (NCS) and electromyography (EMG) were performed. Nerve conduction study (NCS) revealed normal velocity and amplitude of the common peroneal nerve, tibial nerve, sural nerve, and superficial peroneal nerve. Hoffmann reflex, pudendal evoked potential were within normal limits. Electromyography (EMG) showed abnormal spontaneous activities, which are denervation potentials, in the S2-innervated intrinsic foot muscles and the S1-S2 nerve root innervated muscles such as the soleus, gastrocnemius, and lumbar paraspinalis muscle (Table 1) [7–9]. The amplitude of the abnormal spontaneous activities were about 100 μV, indicating that the development of muscle membrane instability following neural injury occurred within 1 month [10] (Fig. 3). Electrodiagnostic results along with the patient’s clinical presentation and MRI findings led us to a diagnosis of left S2 radiculopathy.

**Table 1:** Result of electromyography

Muscle	1 month			3 month			6 month		
	ASA	MUAP	Int. P	ASA	MUAP	Int. P	ASA	MUAP	Int. P
TA	–	Normal	CIP	–	Normal	CIP	–	Normal	CIP
PL	–	Normal	CIP	–	Normal	CIP	–	Normal	CIP
AH	1 + PSW	Normal	PIP	2 + PSW	Polyphasic	PIP	–	Polyphasic	PIP
ADM	1 + PSW	Polyphasic	PIP	1 + PSW	Polyphasic	PIP	–	Polyphasic	PIP
GCN	1 + PSW	Normal	PIP	1 + PSW	Polyphasic	PIP	–	Polyphasic	PIP
Soleus	–	Polyphasic	CIP	1 + PSW	Polyphasic	PIP	–	Polyphasic	PIP
SM	–	Polyphasic	CIP	–	Polyphasic	PIP	–	Polyphasic	PIP
GM	–	Normal	CIP	–	Normal	CIP	–	Normal	CIP
VM	–	Normal	CIP	–	Normal	CIP	–	Normal	CIP
LP	1 + PSW			1 + PSW			–

The patient took pregabalin 75 mg two times per day to control the pain, and after 3 months of medication, the patient’s pain improved from VAS 7 to 5 [11]. A follow up nerve conduction study (NCS), electromyography (EMG) and pelvic magnetic resonance image (MRI) were performed 3 months after onset. Consistently, nerve conduction study (NCS) and Hoffmann reflex were within normal limits, and abnormal spontaneous activities were observed in S2 nerve root innervated muscles. In the pelvic magnetic resonance image (MRI), little residual enhancement was still present along the left S2 nerve root (Fig. 2c, d).

**Fig. 4:**
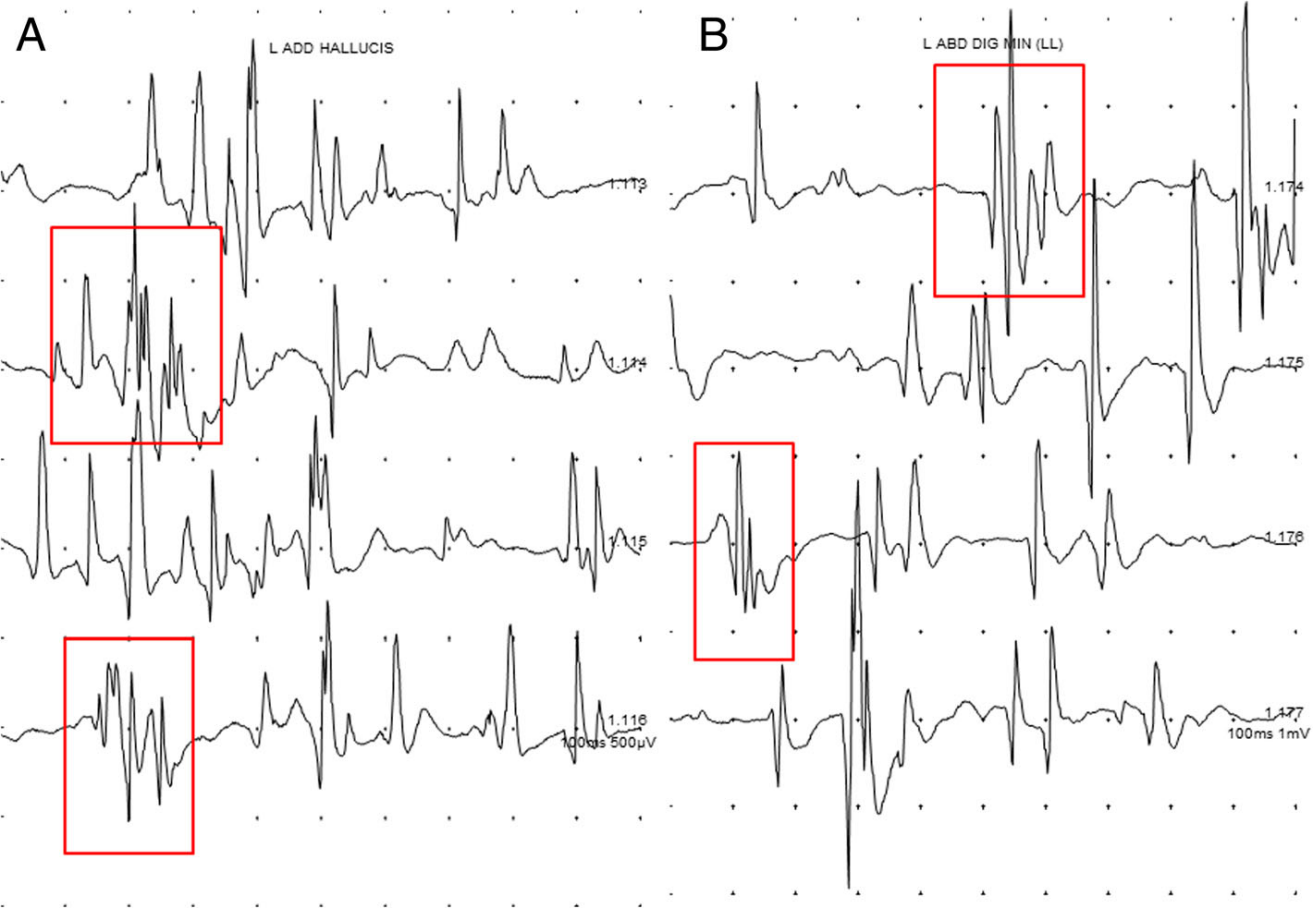
Motor unit action potential (MUAP) on foot intrinsic muscles at 6 month (**a**. left adductor hallucis, **b**. left abductor digiti minimi) since symptom onset. Motor unit action potentials (MUAPs) of polyphasic pattern are observed, indicating recovery state

At 6 month follow up, visual analogue scale (VAS) further improved to VAS 3, and electromyography (EMG) showed motor unit action potentials (MUAPs) of re-innervation pattern instead of abnormal spontaneous activities, indicating recovery state (Table 1, Fig. 4). The patient took pregabalin for a total of 8 months. After that, the patient stopped medication. One year later, the patient’s pain was reduced to a level that was not inconvenient, and we did not prescribe any further medication. Additional nerve conduction study (NCS), electromyography (EMG), and magnetic resonance image (MRI) were not performed.

## Discussion and conclusions

Neuropathic pain in the S2 dermatome occurred in our patient 2 days after bone marrow harvesting. T1 and T2 weighted images of the pelvis magnetic resonance image (MRI) revealed patchy edematous change with enhancement in the sacrum, retrosacral muscles, subcutaneous layer, and around the S2 neural foramen, which appeared to be signs of inflammation [5, 6] (Fig. 2a, b). Nerve conduction study (NCS) was within normal limits performed 4 weeks after onset. However, in the electromyography (EMG), foot intrinsic muscles, gastrocnemius, soleus and lumbar paraspinalis muscle that were innervated by the S1-S2 nerve root showed muscle membrane instability indicating neural injury (Table 1). Based on the patient’s clinical presentation, image studies, and electrodiagnostic studies, we diagnosed the patient with S2 radiculopathy.

Considering the clinical progress and work up results, we suggest mechanical sacral root damage that occurred during puncture needle insertion as the cause of nerve irritation and inflammation. In addition, the possibility of nerve root inflammation by bone marrow leaking into the neural foramen when aspirating the bone marrow should also be considered [12, 13]. There was no suspicious evidence of hematoma around the peripheral nerve, especially the sciatic nerve, located in the pelvic area and the needle’s trajectory appeared too medial and deep on the MRI image. In physical examination, all dermatome sensory were intact. Sensory nerve action potentials showed normal findings, and electromyography (EMG) revealed abnormal spontaneous activity in S2 innervated muscles (foot intrinsic muscle). Therefore, pre-nerve root ganglionic injury is suspected. S2 radiculopathy, which is the damage of the nerve root level, is valid [14].

Our patient took pregabalin for pain control [11]. The patient was followed up for 1 year, and the visual analogue scale (VAS) score that was initially 7 points improved to 1 point. However, because nerve root injury can present with various prognoses depending on the severity, long term follow-up is required for comparison if a same case develops.

We conducted nerve conduction studies (NCS) and electromyography (EMG) for specific neurologic diagnosis. Electromyography (EMG) is especially a useful tests to measure the progression and prognosis of nerve injuries. It is important to measure spontaneous activity and motor unit action potential (MUAP) to characterize nerve injury lesions. Spontaneous activity is the electrical activity recorded by a needle electrode placed in a relaxed muscle. Motor unit action potential (MUAP) is the electrical activity typically recorded by a needle electrode placed in a voluntarily contracting muscle. According to neurophysiologic changes, lesions that are less than one week old are likely to be normal in electromyography (EMG). Therefore, it is recommended to perform the study 3 to 4 weeks after the nerve injury [10]. Abnormal spontaneous activity was presented after more than 3 weeks and may persist up to 3 months. If the lesion is no longer worsening, abnormal spontaneous activity would show low amplitude of less than 100 μV, or abnormal spontaneous activity will not be present after 3 months, Motor unit action potential of the polyphasic pattern would be observed [14]. It is valuable to consider additional neurophysiologic evaluation as a useful study when nerve injury is suspected.

In our case, electromyography (EMG) results performed at 1 month and 3 months after neurological symptoms showed the amplitude of abnormal spontaneous activities of about 100 μV, 300 μV each (Fig. 3). After 6 months, abnormal spontaneous activity was not observed and polyphasic motor unit action potentials (MUAP) were observed (Fig. 4). These findings indicate that the nerve injury is in a recovery state that is no longer worsening and that the patient’s symptoms have improved.

A few studies present sacral nerve root injury. Common causes of sacral root injury are Tarlov cyst [7] and disc rupture [8]. One study reported as iatrogenic injury described a case of sacral nerve root injury after trans-sacral epiduroscopic laser decompression [9]. Our case is a unique sacral nerve root injury case after bone marrow harvesting which has not been previously reported.

Bone marrow harvesting was performed on the patient in the prone position using posterior superior iliac spine (PSIS) puncture [2]. Bone marrow harvesting is generally considered a relatively safe procedure with rare complications, but complications such as aspiration site pain, anemia, vasovagal reaction, and infection have been reported [1, 3]. This case reveals sacral nerve root injury (S2 radiculopathy) after bone marrow harvesting, which to our knowledge, has not yet been reported.

The donor’s safety is most important when conducting bone marrow harvesting. Puncture site positioning and needle manipulation are crucial to prevent side effects such as our case report. The posterior superior iliac spine (PSIS) is the most common collection area because of its prominence, easy access, and thick bone wall. Palpate the posterior superior iliac spine (PSIS) and the prominence of the posterior iliac crest. Mark the locations by drawing an outline using a surgical marking pen. Outline the lateral edge of the lumbar sacral spine as well. This landmark identification procedure is generally effective.

To avoid nerve and vessel injury, aim the needle at about 30° lateral from the para-sagittal plane and 20–25° inferior from the transverse plane when inserting the puncture needle. The exact depth of optimal advancement varies somewhat, but generally, the needle should not be advanced more than 6 cm. If it is unstable, remove the needle and reinsert [15–17]. In our case, puncture needle was inserted too medial from the para-sagittal plane and advanced too much.

Ultrasonography and fluoroscopy imaging are widely used for safe and accurate procedures. Several studies reported that procedures using ultrasonography and fluoroscopy reduced the risk of procedure failure and trauma [18–20]. However, there was no report that bone marrow harvesting was performed using ultrasonography or fluoroscopy. Because bone marrow harvesting is conducted as a blind procedure, It is important to consider how to perform it more accurately and safely if there is a possibility of injury to the donor as in this case. Therefore, we thought it may be helpful to use real-time images with either ultrasonography or fluoroscopy during bone marrow harvesting for the safety of the donor.

Bone marrow harvesting is considered to be a safe procedure with rare complications. However, rare complications of nerve damage may occur as in this case. Clinicians should consider the possibility of sacral nerve root injury and perform bone marrow harvesting with accurate anatomy knowledge and carefulness. Using real-time imaging methods such as ultrasonography or fluoroscopy during the procedure is considered to be a good method for safety.

## Abbreviations

EMG: Electromyography
HSC: Hematopoietic stem cell
HSCT: Hematopoietic stem cell transplantation
MRI: Magnetic resonance image
MUAP: Motor unit action potential
NCS: Nerve conduction study
NSAID: Non-steroidal anti-inflammatory drug
PSIS: Posterior inferior iliac spine
VAS: Visual analogue scale
